# Using Acceleration Data to Automatically Detect the Onset of Farrowing in Sows

**DOI:** 10.3390/s18010170

**Published:** 2018-01-10

**Authors:** Imke Traulsen, Christoph Scheel, Wolfgang Auer, Onno Burfeind, Joachim Krieter

**Affiliations:** 1 Livestock Systems Group, Department of Animal Science, Georg-August University, Albrecht-Thaer Weg 3, 37075 Göttingen, Germany; 2 Institute of Animal Breeding and Husbandry, Christian-Albrechts-University, Olshausenstr, 40, 24098 Kiel, Germany; cscheel@tierzucht.uni-kiel.de (C.S.); jkrieter@tierzucht.uni-kiel.de (J.K.); 3 MKW Electronics GmbH, Jutogasse 3, 4675 Weibern, Austria; wolfgang.auer@smartbow.at; 4 Chamber of Agriculture Schleswig-Holstein, 24327 Blekendorf, Germany; oburfeind@lksh.de

**Keywords:** farrowing, acceleration measurement, CUSUM control chart, management assistance, ear sensor

## Abstract

The aim of the present study was to automatically predict the onset of farrowing in crate-confined sows. (1) Background: Automatic tools are appropriate to support animal surveillance under practical farming conditions. (2) Methods: In three batches, sows in one farrowing compartment of the Futterkamp research farm were equipped with an ear sensor to sample acceleration. As a reference video, recordings of the sows were used. A classical CUSUM chart using different acceleration indices of various distribution characteristics with several scenarios were compared. (3) Results: The increase of activity mainly due to nest building behavior before the onset of farrowing could be detected with the sow individual CUSUM chart. The best performance required a statistical distribution characteristic that represented fluctuations in the signal (for example, 1st variation) combined with a transformation of this parameter by cumulating differences in the signal within certain time periods from one day to another. With this transformed signal, farrowing sows could reliably be detected. For 100% or 85% of the sows, an alarm was given within 48 or 12 h before the onset of farrowing. (4) Conclusions: Acceleration measurements in the ear of a sow are suitable for detecting the onset of farrowing in individually housed sows in commercial farrowing crates.

## 1. Introduction

The course of parturition sets the basis for an optimal start into the suckling period, both for the sow and their piglets. Problems during parturition often result in health disorders or fertility problems and consequently economic losses for the farmer [1]. Continuous animal surveillance before and during farrowing allows rapid and early human attendance to the sow and their piglets if necessary. Under practical farming conditions continuous animal surveillance is unrealistic and not feasible as it requires high personal effort and is too costly. Automatic monitoring systems are a beneficial management tool to address this issue. Associated alarm systems to predict the onset of farrowing enable targeted work assignments for human resources. Monitoring systems are mainly based on changes in social as well as activity behavior of animals before the onset of farrowing. In several studies an obvious increase in sows’ activity during the last 24 h before farrowing is demonstrated (for example, [2,3,4,5]). Increased activity is mainly due to nest building behavior [6,7]. The means of predicting the onset of farrowing activity was examined in several studies using photocell systems [2,4,8], force sensors [2], or acceleration sensors combined with different analysis algorithms such as dynamic generalized linear models, CUSUM charts, and hidden Markov models [5,9]. In the preceding literature, the specific task of determining the onset of farrowing from acceleration data was formulated in the framework of hidden state space models. This was done either through specifying a hidden Markov model or a dynamic linear model (DLM) [9,10]. Generally, the DLM aims to produce a smoothed approximation of the observables, that is, the observed data. This is implemented by having a stochastic process of unobservable, latent variables or hidden states drive the output of a linear filter so as to produce the smoothed observable process as prescribed by the Kalman equations [11]. The fit is successively calculated using a transition matrix to guide the evolution of the hidden process itself and a design matrix to draw the next predicted value of the smoothed data. The design matrix enables a representation of the smoothed data as a superposition of components. In Manteuffel et al. [12], a seasonal and a trend component were specified, while Pastell et al. [11] used a split into seasonal, slope and trend components. The trend components were then further used to produce a farrowing alarm under the application of CUSUM charts.

The aim of the present study was to predict the onset of farrowing measuring sows’ activity using acceleration measurements from ear sensors. A simple and yet possibly more robust approach is presented. The method uses a cancellation approach on discretized feature sequences, where 24 h offset differences were cumulated to produce a CUSUM-tractable trend without having to rely on a parametric fitting procedure.

## 2. Materials and Methods

The onset of farrowing was to be predicted by monitoring sows’ activity behavior using acceleration measurements. As references, video recordings were used to determine the onset of farrowing. To implement an early warning system for the onset of farrowing, a classical CUSUM chart using different parameters based on the sensors’ acceleration measurements was set up.

### 2.1. Animals and Housing

Data was acquired in three batches in one compartment of the farrowing unit at the Futterkamp research farm of the Chamber of Agriculture in Schleswig-Holstein, Germany, from December 2013 to March 2014. Sows were housed in commercial farrowing pens (2 m × 2.6 m) with farrowing crates. Sows received a commercial lactating meal (13.4 MJ of ME per kg feed). Seven days before the expected date of farrowing, sows were washed and moved into the farrowing pen. Animal surveillance was conducted regularly between 5 a.m. and 9 p.m. One day before the expected date of farrowing, piglet resting areas were heated with a heat lamp and floor heating. Farrowing was induced starting at Gestation Day 115. All sows were videotaped with color cameras (Santec, VTC-249/IRP/W or VTC-279IRPWD) 24 h a day from being housed until two days after farrowing. For analysis purposes, data of 20 cross-bred sows with parities ranging between 1 and 11 with an average of 4.4 were available.

### 2.2. Sensor Systems

**Figure 1:**
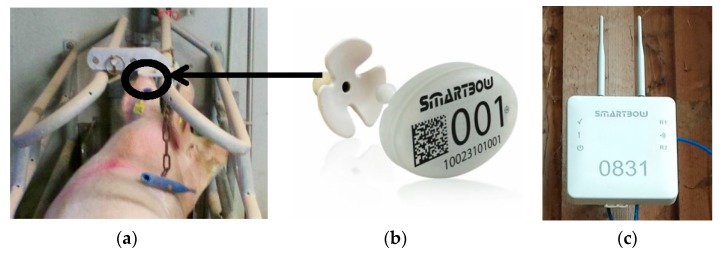
Sows were equipped with an ear tag (**a**,**b**) including an accelerometer. The tag actively sent their information to the receivers (**c**).

The sensor system, provided by MKW Electronics GmbH based in Weibern (Austria), consisted of the ear tags, wall mountable receivers, and computing infrastructure for synchronizing and integrating the received data. An ear tag (Figure 1a,b) had a weight of about 35 g and incorporated a 3-D accelerometer measuring or sampling acceleration values along the three internal axes (*x*, *y*, and *z*). The ear tags sent each of the three measurements (that is, *x_t_*, *y_t_*, *z_t_*) at time *t*, actively, autonomously, and unidirectionally to any of the four installed receivers. The predefined sampling rate of 1 Hz for the present data set resulted from limitations in battery life. Higher sampling rates (5 or 10 Hz) reduced battery life enormously, while sampling rates lower than 1 Hz reduced analysis accuracy. Further information on the sensor system can be found in [13].

### 2.3. Acceleration Transformation Procedure

On average, 74,619 of 86,400 possible acceleration measurements per day and sow were stored. The time-series s of total acceleration was calculated from the *x_t_*, *y_t_*, and *z_t_* triples of acceleration at time *t* as follows:

(1) $$s_{t}=\sqrt{x_{t}^{2}+y_{t}^{2}+z_{t}^{2}}.$$

Various distributions (properties/features) of the acceleration were determined for windows w of length 60, 30, and 10 min as observed through the measurements: mean, standard deviation, variance, skewness, kurtosis, median, 25th percentile, 75th percentile, and *p*-variation. The variation was calculated for the 1st, 2nd, and 3rd order (*p*):

(2) $$variation_{p}\left(s(w_{t})\right)=\overset{\#obspwr}{\underset{i=1}{\sum }}{\left|s_{i+1}-s_{i}\right|}^{p}$$

where p=1,2,3, and *#obspwr* is the number of observations in window $w_{t}$ around time t.

**Figure 2:**
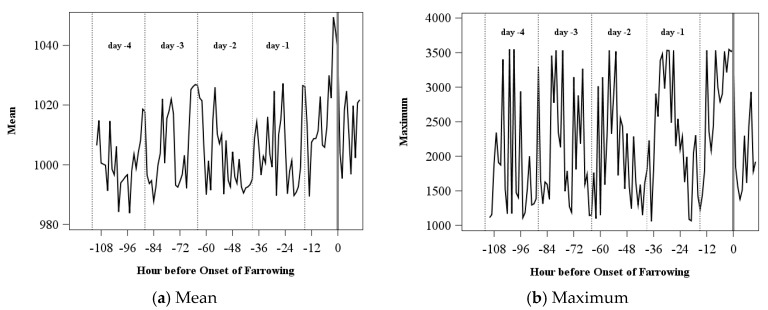

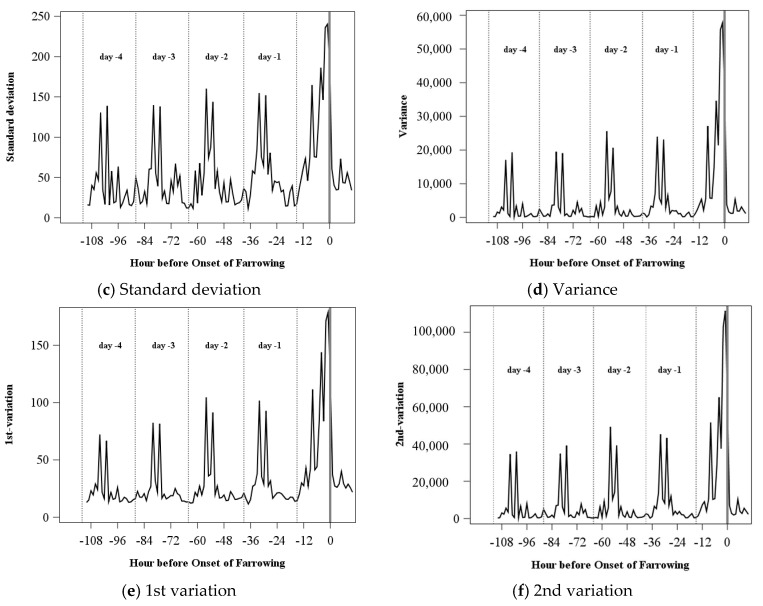
Exemplary distribution characteristics for one sow from Day −4 until onset of farrowing at 2:11 p.m. on Day 0 (corresponds to Hour 0 in the figures) for one sow.

An example of the variance of the acceleration signal for one sow can be found in Figure 2d. The circadian rhythm and the increase before the onset of farrowing (Hour 0), which is still higher than the maximum of the days before, are clearly visible. The same can be seen for the distribution characteristics standard deviation, and the 1st, 2nd, and 3rd variation (for example, Figure 2e,f). All other characteristics showed no regular pattern over time (examples of mean and maximum are given in Figure 2a,b) and were excluded from further analysis.

A smooth series with a clearly defined expectation, with low noise and random variation, is beneficial for obtaining good results of a control chart. Two further transform steps were applied to the distribution sequences. To smooth the signal, multiple acceleration indices were calculated for each of the distribution characteristics variation, standard deviation, and 1st, 2nd, and 3rd variation. To avoid false positive alarms indicating the circadian rhythm, the difference and quotient of the average acceleration characteristic within one time period between consecutive days were calculated:

Diff: the difference between the average of the acceleration characteristic of a specific time interval *t* at day *d* and the moving average of the same time interval *t* the day before *d* − 1, where the moving average was calculated for ±1, 3, and 5 time intervals for the 60 min period, ±1, 3, 5, and 9 time intervals for the 30 min periods, and ±1, 5, 9, 13, 19, and 25 time intervals for the 10 min periods; for example, a 60 min period difference between the acceleration characteristic of the period from 6 to 8 a.m. and (a) ±1: the average of the period from 5 to 7 a.m., (b) ±3: the average of the period from 3 to 10 a.m., and (c) ±5: the average of the period from 1 to 12 a.m.

Quot: the quotient of the average of the acceleration characteristic of a specific time interval *t* at day *d* and the moving average of the same time interval *t* the day before *d* − 1, where the moving average was calculated for ±1, 3, and 5 time intervals for the 60 min periods, ±1, 3, 5, and 9 time intervals for the 30 min periods, and ±1, 5, 9, 13, 19, and 25 time intervals for the 10 min periods.

Over: an extension of Quot (time intervals chosen analogously) with consideration of time overlaps between hours to take shifts of activity within short time intervals into account, calculated as a quotient of the average of the acceleration characteristic of a specific time interval *t* at day *d* with a certain time overlap and the moving average of the same time interval *t* with overlap the day before d − 1, where the moving average was calculated for the intervals described above with a 10 min overlap for the time period of 60 min, a 5 min overlap for the time period of 30 min, and a 2 min overlap for the time period of 10 min; for example, a 60 min period ratio between the acceleration characteristic of the time period from 6 to 7 a.m. was calculated between the time period from 5:50 to 6:10 a.m. and (a) ±1: the average of the period from 4:50 to 8:10 a.m., (b) ±3: the average of the period from 2:50 to 10:10 a.m., and (c) ±5: the average of the period from 0:50 a.m. to 12:10 p.m.

**Figure 3:**
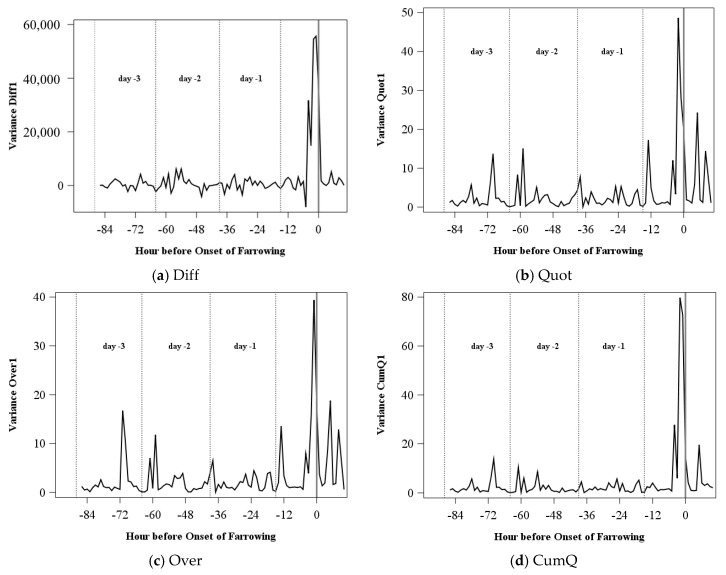

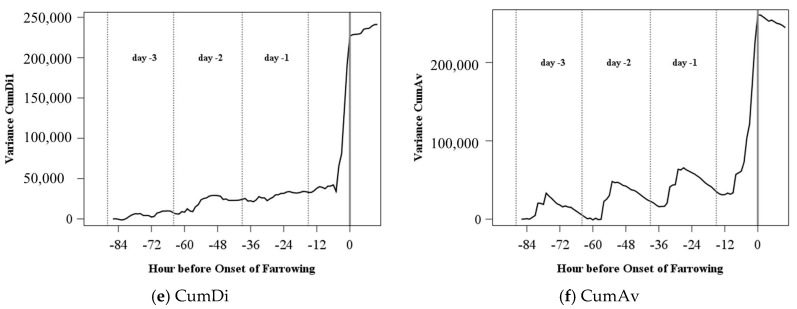
Exemplary acceleration indices of one sow based on the distribution characteristic variance for a time period of 60 min and a range of the moving average of 1 for one sow.

An example for each of the indices is shown in Figure 3a–c. The index Diff is centered on zero and corrects for the individual activity level of the sow. The ratio Quot is a much smoother index and has only positive values. In Over, the high peaks are eliminated but the increase before the onset of farrowing stands out.

However, the resulting acceleration indices still showed substantial variation. The indices series should obey a cancellation property whenever days are similar to each other (as would be the case when a sow exhibits a similar behavior at about the same time on each day). Cumulative sums were tested for a further smoothing of the acceleration signal:

CumDi: for time interval *t* at Day *d* calculated as the cumulative sum of Diff up to time interval *t* at day *d*.

CumQ: quotient of the average of the acceleration characteristic of a specific time interval *t* at day *d* and the moving average of the cumulative sum up to the same time interval *t* the day before *d* − 1.

CumAv: for time interval *t* at day *d* calculated as cumulative sum of difference between the average of the acceleration characteristic of a specific time interval *t* at Day *d* and the average over the last 24, 48, or 144 time intervals depending on the time period of 60, 30, or 10 min.

Figure 3d–f shows examples for the cumulative sum acceleration indices. The index CumDi still shows much variation. For this exemplarily sow, particularly at the days after being housed, high variation can be found. The signal of the index CumQ is much smoother. The index increases over time, and it increases enormously just before the onset of farrowing. In the last index presented (CumAv), the circadian rhythm is not eliminated. However, the index ranges on a much smaller level than it did before the onset of farrowing.

**Figure 4:**
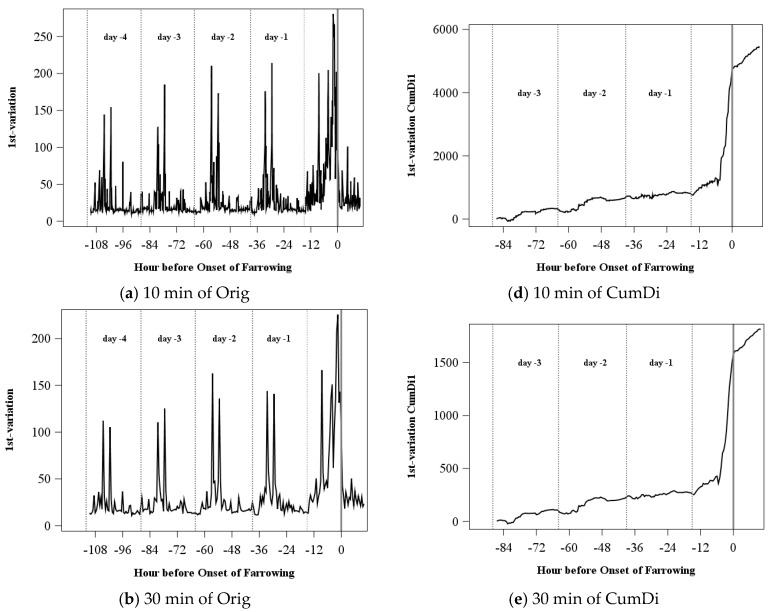

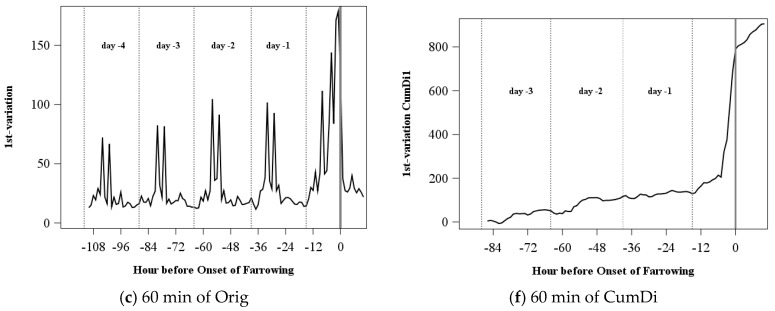
Time intervals 10, 30, and 60 min exemplary for the original values (Orig: **a**,**b**,**c**) and the acceleration index CumDi (**d**,**e**,**f**) for the distribution parameter 1st variation with a range of the moving average of 1 for one sow.

In Figure 4, the different time periods (10, 30, and 60 min) are compared using the original values and the acceleration index CumDi of the distribution characteristic 1st p-variation. The longer the time period the smaller the changes of the original values are (Figure 4a–c). However, maximum peaks and the increase before farrowing can be found for all time periods. The acceleration index CumDi (Figure 4d–f) shows a similar course for the 10 and 30 min periods, although the 10 min period has some smaller changes. For the acceleration index for 60 min, the day-to-day variation is higher and the increase before the onset of farrowing starts later.

**Figure 5:**
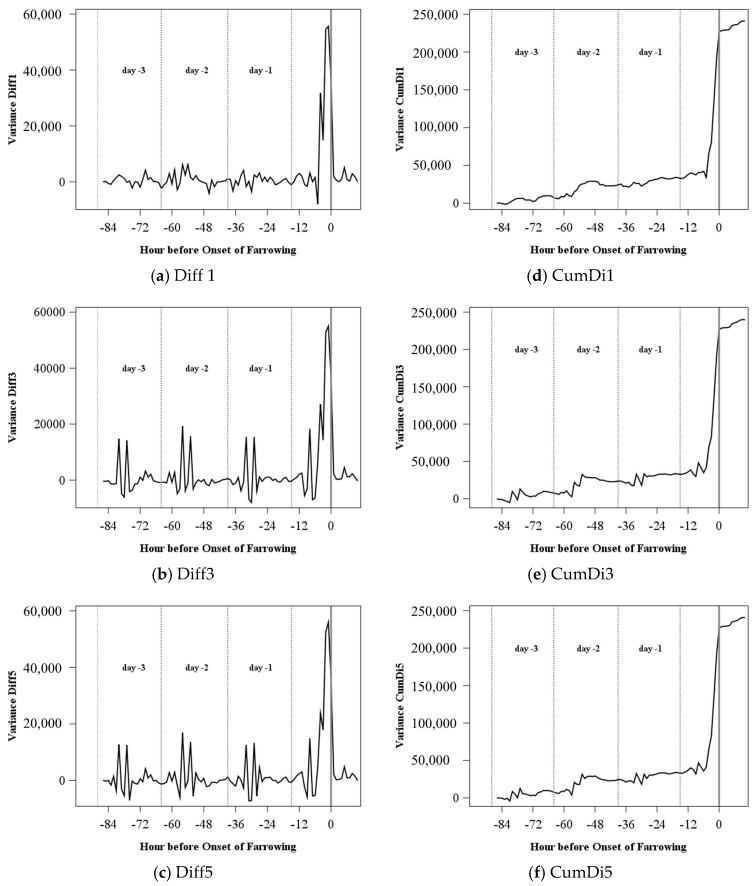
The range (1, 3, 5) for the moving average in acceleration indices Diff (**a**,**b**,**c**) and CumDi for 60 min period (**d**,**e**,**f**) of the distribution characteristic variance for one sow. Note that 0 indicates the onset of farrowing.

In Figure 5, the influence of the different ranges of the moving average in the acceleration indices are shown exemplarily for the indices Diff and CumDi of the distribution characteristic variance for the 60 min period. Increasing the range of the moving average from the ±1 time interval to ±5 time intervals resulted in more fluctuations in the course of both indices.

### 2.4. CUSUM Control Charts

*General Aspects:* To predict the onset of farrowing, defined as the birth of the first piglet, a CUSUM (CUmulative SUM) control chart [14] was applied. In general, control charts deliver an alarm if a monitored process or trait deviates from a predefined target value, that is, the process is out of control. In CUSUM control charts, positive and negative deviations are summed up (cumulative sum) separately. In the present study, the acceleration indices per time interval are monitored using a one-sided CUSUM chart only for positive deviations. The cumulative sum (*C*^+^) for the acceleration index (*X*) at time point *i* is calculated according to the following formula:

(3) $$C_{i}^{+}=\max\left[0,\;X_{i}-\left(\mu_{0}+k\right)+C_{i-1}^{+}\right].$$

With $\mu_{0}$ indicating the sow individual average acceleration, *k* the allowance value, and $C_{i-1}^{+}$ the cumulative sum at the preceding time point. If the cumulative sum exceeds the control limit (CL), an alarm is given. The control limit is calculated as the product of the sow individual standard deviation of the acceleration index and a smoothing value (h).

*Parameterization:* For the control chart, data of 24 sows could be used. The time period from housing-in into the farrowing pen to the calculated day of farrowing is divided into a parameterization period and a prediction period. Days −5 and −4 before the calculated farrowing date were defined as the parameterization period. Acceleration measurements within this period were used to parameterize the control chart in three different scenarios: data from (1) Day −4, (2) Days −4 and −5, (3) Days −5. Within the prediction period (Days −3 until Day 0), alarms for the onset of farrowing are expected.

*Evaluation*: For evaluation purposes of the CUSUM control chart, the point in time of an alarm was measured relative to the real onset of farrowing for each sow (date and time in hours and minutes) and was defined by birth of the first piglet. The point in time of the birth of the first piglet was determined manually using video recordings. Note that the calculated day was not taken into account for the evaluation. The first exceedance of the upper control limit by the CUSUM chart was considered as an alarm. Different values for the parameter *k* and *h* were compared: allowance value k: 0.1, 0.25, 0.5, 1, 1.5, …, 12.5, 15, 20, 25, 30, and smoothing value *h*: 4, …, 10.

In summary, the following influence factors on the performance of the CUSUM chart were evaluated:

- Distribution characteristic: standard deviation, variance, and variation of 1st, 2nd, and 3rd order.
- Acceleration index: Orig, Diff, Quot, Over, CumDi, CumQ, CumAv.
- Time period: 10, 30, 60 min.
- Interval of moving average, depending on time period (10 min: 1, 5, 9, 13, 19, and 25; 30 min: 1, 3, 5, and 9; 60 min: 1, 3, and 5).
- Allowance value *k*: 0.1, 0.25, 0.5, 1, 1.5, … 12.5, 15, 20, 25, and 30.
- Smoothing value *h*: 4, …, 10.
- Parameterization period (Day −4, Day −5, and Days −4 and −5).

## 3. Results

In general, the time and the parameterization period, different ranges of the moving average interval, and the distribution characteristic revealed only small changes in the performance of the CUSUM chart. The detection rate of the onset of farrowing depended mostly on *k* and *h* values as well as the type of acceleration index.

Table 1 shows the maximal detection rate for the onset of farrowing within 12 or 48 h before the onset of farrowing per distribution characteristic, acceleration index, and time period. Summarizing all scenarios, 45–84.2% of the sows could be detected within 12 h before the onset of farrowing and 68.4% up to 100% within 48 h. Best results, both for the detection rate 12 and 48 h before the onset of farrowing, were found for the acceleration characteristic CumDi of the distribution characteristic 1st variation and the time period of 60 min, where 84.2% or 100% of the sows were detected. Comparing all indices and distribution parameters, most sows were detected with the time window of 60 min. The lowest detection rates were observed for the original signal, while the indices including a cumulative sum performed better. In detail, the acceleration index Diff led to better results than Quot, and CumDi led to better results than CumQ. Differences between Over and Quot were small, and no obvious trend could be found.

The influence of the different ranges of the moving average interval in calculating the acceleration indices and of the parametrization period on the maximal detection rate is shown in Table 2.

The distribution characteristic 1st variation and the 10 min period was chosen, as for this time period the largest number of ranges for the moving average (1, 5, 9, 13, 19, and 25) were present. Increasing the range of the moving average (from 1 to 25) increased the detection rate by 5.6–26.4%, except for CumDi for which the detection rate for the parametrization period of Days −4 or −5 was almost constant (0–5.5%) and for Days −4 and −5 even decreases (−5.6%). However, as mentioned before the acceleration index, CumDi showed the best performance, with a detection rate of almost 80%. Differences between parametrization periods were only small, but if differences were present, the highest detection rates were found for the CUSUM chart parametrized with data of Day −4.

**Figure 6:**
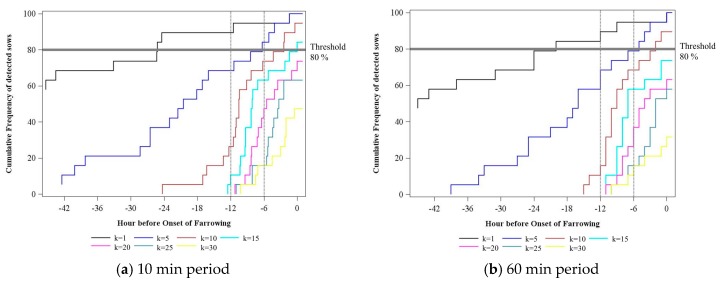
Cumulative sum of the number of sows detected within the whole prediction period exemplarily for the 10 and 60 min period. Time window from Hour −6 to Hour −12 is marked.

Figure 6 shows the cumulative detection rate for the acceleration characteristic CumDi of the distribution characteristic 1st variation and the time period of 10 and 60 min depending on different *k* values. Choosing a *k* value of 1 could detect all sows up to the onset of farrowing, but only 10.5% of them within 12 h before the onset of farrowing. With an increase in the *k* value, the percentage of sows found within 12 h before the onset of farrowing increased. However, at the same time, the total number of sows detected decreased. For example, using the 10 min time period, with a *k* value of 30, all alarms were given within the 12 h window, but only 47.4% of the sows were detected. In this example, if a *k* value of 15 was used, 84.2% of the sows were detected, only one of which was outside the 12 h window before the onset of farrowing.

**Figure 7:**
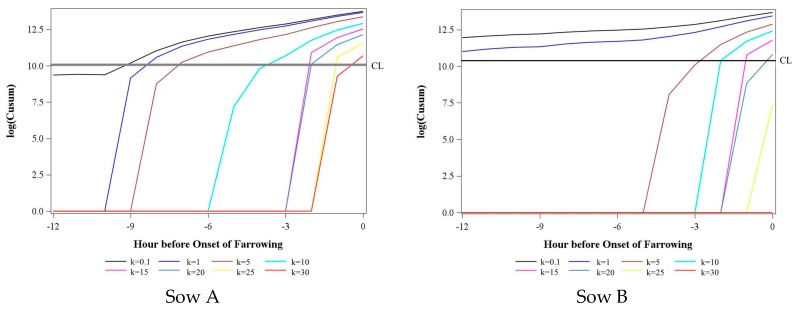
Influence of the *k* value on the alarm time (Chart exceeds control limit (CL)) of the CUSUM chart (*h* = 4) exemplarily for two sows and the acceleration index 1st variation and the distribution characteristic CumDi with the time interval of 60 min and a range of the moving average of 1. Note that, for a better presentation, the CUSUM statistic is transformed on a log scale.

Variability between CUSUM charts of different sows is shown in Figure 7. Exemplarily for two sows, CUSUM charts with *h* = 4 and different *k* values for the distribution characteristic 1st variation of the acceleration index CumDi with the time interval of 60 min were plotted. Different *h* values are not shown, as results were very similar. For both sows, findings of Figure 6 could be confirmed. If the *k* value increased, the control limit of the alarm was given later (exceeding upper control limit), that is, closer to the onset of farrowing. However, Figure 6 shows a large difference between the sows in the exact time points. Choosing a *k* value of 5 resulted in an alarm six or three hours before the onset of farrowing for Sow A and B, respectively. Sow behavior was individual.

## 4. Discussion

Best performance of the algorithms required a statistical distribution characteristic that represented fluctuations in the signal (for example, 1st variation) combined with a transformation of this parameter by cumulating differences in the signal within certain time periods from one day to another. Results indicated that this transformation procedure mainly improved the detection rate of the CUSUM chart. The length of the time period (10, 30, and 60 min) was of minor importance. Smaller periods reflected more details in the signal, but this was not beneficial for the detection algorithm. To reduce the calculation effort for practical implementations, a 60 min period could be recommended. Calculating a difference between days was superior to a quotient, that is, the level of the activity had a smaller influence on the result than the absolute difference. Of great importance was the accumulation element in the transformation algorithm. If differences between days increased, the cumulative sum of the differences increased even more and a single difference reduced the cumulative sum only slightly. The enormous increase in activity of a sow before the onset of farrowing led to a fast increase of the acceleration index CumDi, as large differences to the day before could be measured with the acceleration data. The increase in activity was mainly a result of performing nest-building behavior [6,7]. Compared to loose-housed sows, crate-confined sows perform activities considered as ‘re-directed’ nest building behavior, such as pawing, rooting, or mouthing the crate fixtures [15,16,17]. In the literature, different points in time are described for the beginning of nest-building behavior, but mainly the last 24 h before farrowing are named [2,3,4,5]. This variation in individual behavior can be confirmed with the present data. However, with an optimally calibrated CUSUM chart, the corresponding alarm could be shifted closer to the onset of farrowing.

Data of Days −4 and −5 before the calculated day of farrowing were used for parametrization of the CUSUM chart, as the prediction period started from Day −3. This relatively long prediction period was necessary as 10 sows started farrowing before the calculated date, one even exactly on Day −3. However, the CUSUM chart was able to give an alarm for this sow. The different scenarios for the parametrization period (Day −4, Day −5, or an average of Day −4 and −5) had little influence on the detection rate. In consequence, for practical implementations, a lag of one day before the prediction period would be sufficient. Longer periods are not necessary. Nevertheless, if data from Day −4 are missing, data from Day −5 could be used and would deliver acceptable results as well.

Calibration parameters for the CUSUM charts were the *k* and *h* values. The different scenarios clearly showed that all sows could be detected within 48 h before the onset of farrowing. With a fine tuning of the *k* and *h* values, the point in time of the alarm could be influenced within a certain range. The closer the first alarm was shifted to the onset of farrowing, the fewer sows were detected in total. Fine adjustments also showed sow individual differences in the optimal *k* value if the alarm should be given to a certain point in time. Nevertheless, the overall detection rate was not affected.

As farrowing was induced for all sows, the data set was suitable as a proof of concept for the presented algorithm. For the evaluation of sensitivity and specificity, a larger data set, probably without inducing farrowing, should be used. For practical purposes on the present farm, the time period within 6–12 h before the onset of farrowing was most interesting. An alarm within that time window offered the opportunity for targeted attendance to the sow by stockpersons. Surveillance intervals could be shortened, and the pen could be prepared for an optimal start of the birthing (for example, activating a heating source). A useful alarm should be given within 12 h before the onset of farrowing. However, the alarm time window can be adjusted to farmer’s purposes. A warning system with two alarms was also possible: a first relatively early alarm (six to eight hours before the onset) so that stockpersons could be prepared, and a second alarm very close to the beginning (one to two hours before the onset).

## 5. Conclusions

In conclusion, acceleration measurements at the sows’ ears need to be transformed before being processed in a CUSUM control chart. With the transformed signal, sows could be reliably detected, and fine tuning the alarm could be adjusted for stockpersons’ purposes. However, future studies should focus more on individual differences between sows, as the relatively small number of sows in the present study was not sufficient to determine a systematic influence.

## Figures and Tables

**Table 1:** Maximal frequency of sows detected (%) for the onset of farrowing within 12 or 48 h before the onset of farrowing (first alarm for each sow) per type of acceleration index (Orig, … CumAv), distribution characteristic (Std, …3rd variation), and time period (10, 30, 60 min) for parameterization period of Day −4 independent of the interval length of the moving average and the *k* and *h* values. Bold font indicates the best performance.

Index Time Period		Orig		Diff		Quot		Over		CumDi		CumQ		CumAv	
**N to find**		20		19		19		19		19		19		19	
**Time period**		12 h	48 h	12 h	48 h	12 h	48 h	12 h	48 h	12 h	48 h	12 h	48 h	12 h	48 h
Std	60	70.0	90.0	78.9	94.7	63.2	89.5	63.2	89.5	78.9	100.0	68.4	94.7	73.7	94.7
	30	65.0	85.0	73.7	89.5	57.9	84.2	57.9	84.2	73.7	100.0	63.2	89.5	73.7	94.7
	10	50.0	80.0	63.2	84.2	52.6	78.9	47.4	78.9	73.7	100.0	63.2	84.2	73.7	94.7
Var	60	70.0	90.0	78.9	94.7	52.6	94.7	57.9	89.5	78.9	100.0	73.7	94.7	78.9	100.0
	30	60.0	85.0	73.7	89.5	42.1	78.9	52.6	78.9	78.9	100.0	63.2	89.5	78.9	94.7
	10	50.0	80.0	57.9	84.2	42.1	78.9	42.1	73.7	78.9	100.0	52.6	78.9	78.9	94.7
1st-var	60	60.0	90.0	78.9	94.7	73.7	94.7	73.7	94.7	84.2	100.0	73.7	94.7	73.7	94.7
	30	50.0	85.0	73.7	89.5	68.4	89.5	68.4	89.5	78.9	100.0	68.4	89.5	73.7	94.7
	10	45.0	75.0	57.9	84.2	57.9	89.5	57.9	94.7	78.9	100.0	63.2	89.5	73.7	94.7
2nd-var	60	65.0	90.0	78.9	94.7	57.9	94.7	63.2	94.7	78.9	100.0	68.4	94.7	73.7	94.7
	30	55.0	85.0	68.4	89.5	47.4	84.2	47.4	84.2	78.9	100.0	52.6	89.5	73.7	94.7
	10	50.0	80.0	57.9	84.2	36.8	78.9	42.1	78.9	78.9	100.0	52.6	78.9	73.7	94.7
3rd-var	60	60.0	90.0	68.4	94.7	41.1	84.2	36.8	73.7	78.9	94.7	52.6	89.5	78.9	94.7
	30	55.0	85.0	63.2	89.5	26.3	68.4	31.6	78.9	73.7	94.7	47.4	78.9	73.7	94.7
	10	45.0	85.0	57.9	84.2	31.6	68.4	26.3	68.4	73.7	94.7	42.1	73.7	73.7	94.7

**Table 2:** Maximal detection rate (%) for the onset of farrowing within 12 h before the onset of farrowing (first alarm for each sow) per type of acceleration index (Diff, …, CumQ), parameterization period (m4: Day −4, m5: Day −5, m45 = Days −4 and −5), and interval length of the moving average (Int_x_, x = ±1,5,9,13,19,25) for the distribution characteristic 1st variation and 10 min period independent of the *k* and *h* values.

		Int_1_	Int_5_	Int_9_	Int_13_	Int_19_	Int_25_
Diff	m4	42.1	47.4	52.6	57.9	57.9	57.9
	m5	44.4	44.4	55.6	55.6	55.6	55.6
	m45	44.4	44.4	55.6	55.6	55.6	55.6
Quot	m4	36.8	36.8	47.4	57.9	52.6	57.9
	m5	38.9	44.4	38.9	44.4	50.0	50.0
	m45	44.4	44.4	44.4	50.0	50.0	50.0
Over	m4	31.6	36.8	52.6	52.6	52.6	57.9
	m5	44.4	44.4	44.4	44.4	44.4	50.0
	m45	38.9	44.4	38.9	50.0	50.0	50.0
CumDi	m4	78.9	78.9	78.9	78.9	78.9	78.9
	m5	55.6	55.6	55.6	55.6	61.1	61.1
	m45	72.2	72.2	72.2	66.6	66.6	66.6
CumQ	m4	36.8	36.8	42.1	52.6	63.2	63.2
	m5	44.4	38.9	44.4	55.6	50.0	55.6
	m45	38.9	38.9	44.4	50.0	50.0	55.6
